# Giant left atrium and management modalities (surgical vs. conservative): a case report from Mauritania

**DOI:** 10.1097/MS9.0000000000001132

**Published:** 2023-08-04

**Authors:** Echreiva Med Sidi El Moctar, Chighaly El Hadj Sidi, Mohammed Abdulrazzak, Maher Eldeghedi, Abdelghader Thoraya, Khaled Boye

**Affiliations:** a Faculty of Medicine, Ain Shams University, Cairo, Egypt; b Center National de Cardiology, Nouakchott, Mauritania; c Faculty of Medicine, University of Aleppo, Aleppo, Syrian Arab Republic; d Mansoura Medical School, Mansoura University, Mansoura, Egypt

**Keywords:** case report, giant left atrium, Mauritania, mitral stenosis, rheumatic heart disease

## Abstract

**Introduction and importance:**

Giant left atrium (GLA) is a rare condition often associated with rheumatic heart disease and can lead to cardiac and extracardiac complications. In this case report, the authors present a rare case of GLA with extracardiac complications, highlighting the importance of prompt diagnosis and management.

**Case presentation:**

A 54-year-old woman with a 25-year history of mitral stenosis caused by rheumatic heart disease presented with symptoms of dyspnea, orthopnea, and palpitations. Diagnostic tests revealed an enlarged left atrium, pleural effusion, severe pulmonary hypertension, and tricuspid regurgitation. The patient was treated with diuretics and ACE (angiotensin-converting enzyme) inhibitors and is currently on a medication regimen with regular follow-up appointments.

**Clinical discussion:**

GLA can cause cardiac and extracardiac complications, and conservative treatment and surgery are both involved in the management plan. The reduction of left atrial size by surgery may eliminate symptoms, reduce postoperative complications, and increase the probability of regaining sinus rhythm.

**Conclusion:**

Observational data on managing GLA is limited, and mortality can be high. Cardiovascular surgeons should carefully consider surgical options, and screening and follow-up are essential for early detection and management in patients with long-standing rheumatic heart disease.

## Introduction

Highlights

- Giant left atrium (GLA) is a rare condition often associated with rheumatic heart disease and can lead to cardiac and extracardiac complications.
- A 54-year-old woman with a 25-year history of mitral stenosis caused by rheumatic heart disease presented with symptoms of dyspnea, orthopnea, and palpitations.
- The chest X-ray results indicated cardiomegaly and pleural effusion on the right side.

Giant left atrium (GLA) is a rare cardiac condition that can arise in conjunction with various underlying heart diseases. One of the most closely associated conditions is rheumatic heart disease, which can result in mitral valve stenosis or regurgitation, and GLA is observed in only 3–4% of patients with this disease^1^.

Defining GLA can vary among researchers, but a commonly used criterion is an anteroposterior dimension of 65 mm^2^. This condition can lead to both cardiac and extracardiac complications, making early diagnosis and management crucial^3^.

Diagnostic imaging tests such as chest X-ray and transthoracic echocardiography are used to identify GLA, and transesophageal echocardiography can be employed to determine its dimensions. Computerized tomography (CT) chest scans can also help to identify any extracardiac complications^4^.

In this case report, we present a rare case of GLA with extracardiac complications, highlighting the importance of prompt diagnosis and management. Further research is needed to better understand the underlying mechanisms and risk factors associated with this condition.

## Case presentation

A 54-year-old woman visited the cardiology department with symptoms of dyspnea (classified as grade VI by the New York Heart Association), orthopnea, and palpitations which had been occurring for the past 4 months. She also reported recurrent chest infections, but did not experience hoarseness or difficulty swallowing. There was no pertinent medical history in her family, but she had a 25-year history of mitral stenosis caused by rheumatic heart disease. Twenty years ago, the patient underwent open-heart surgery and mitral valve valvuloplasty to address the condition.

During the physical examination, the patient’s blood pressure was 110/90 mmHg, respiratory rate was 14, heart rate was 106 beats per minute, and temperature was 37°C. Upon conducting a cardiac examination, it was observed that the apex of the heart was located between the fourth and fifth rib on the midclavicular line. Dilated jugular veins were observed, but there was no lower limb edema. A systolic murmur was detected, which was attributed to mitral and tricuspid regurgitation.

**Figure 1:**
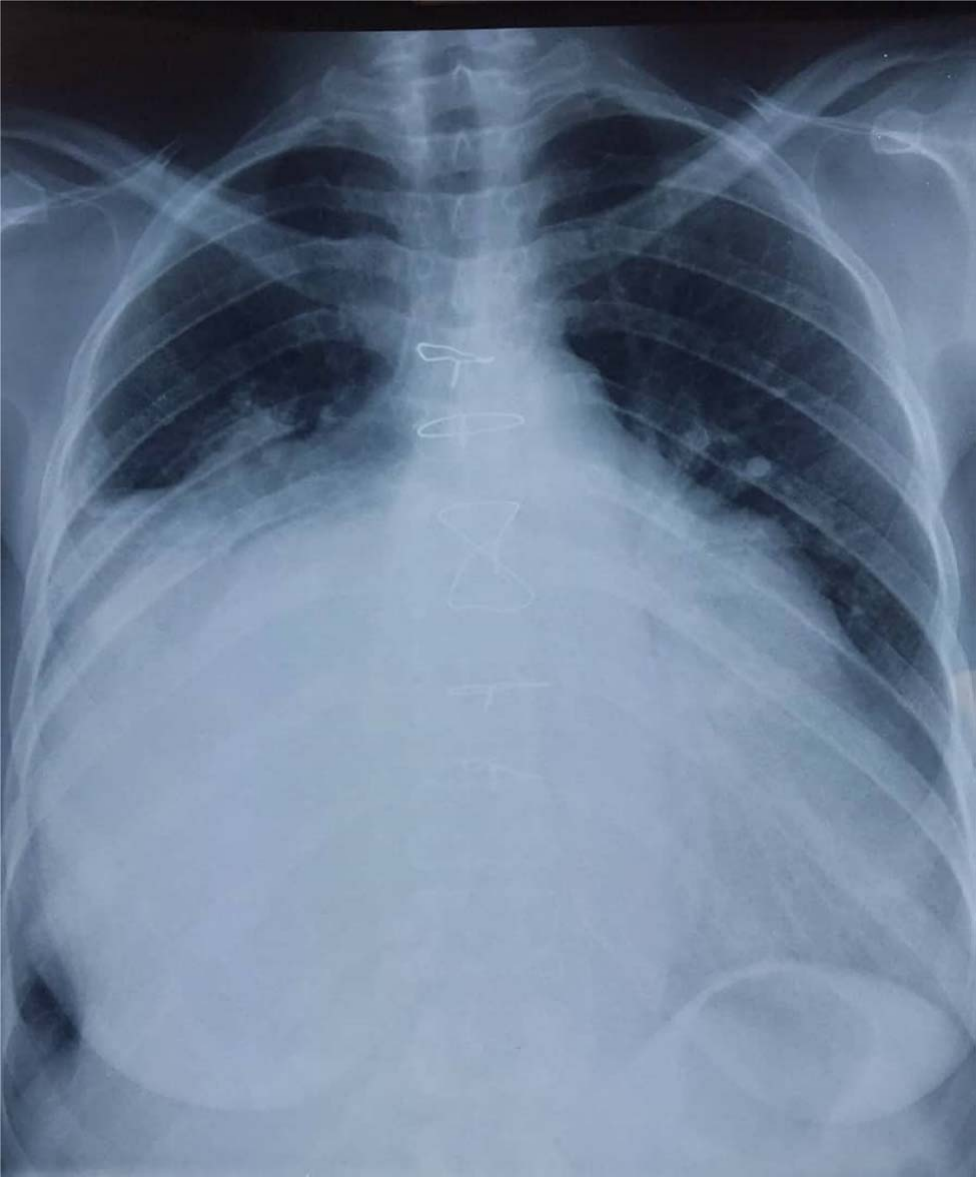
X-ray, increase the cardiothoracic index (cardiomegaly), subdiaphragmatic apex, medium convex left arch, and pleural effusion on the right side.

**Figure 2:**
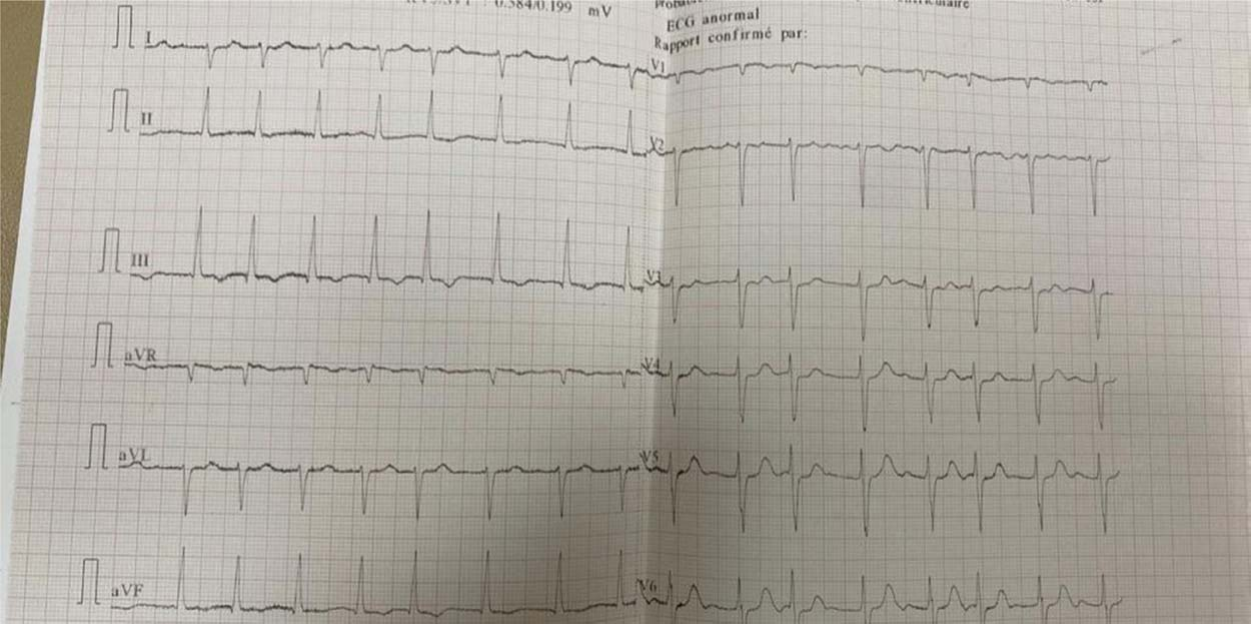
The ECG shows atrial fibrillation along with left axis deviation with an average rate of 98 bpm.

**Figure 3:**
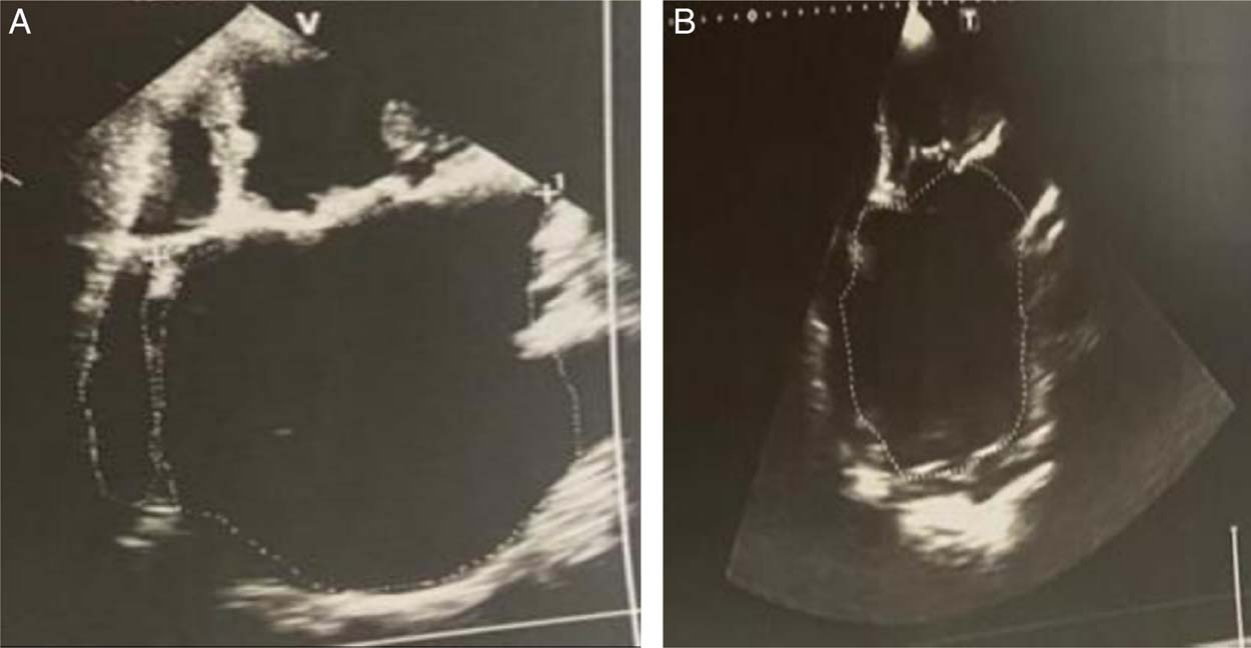
(A) Apical two-chamber window showing a giant left atrium at 130 cm^2^. (B) Apical four-chamber window showing a giant left atrium with a size of 165 cm^2^ in area.

**Figure 4:**
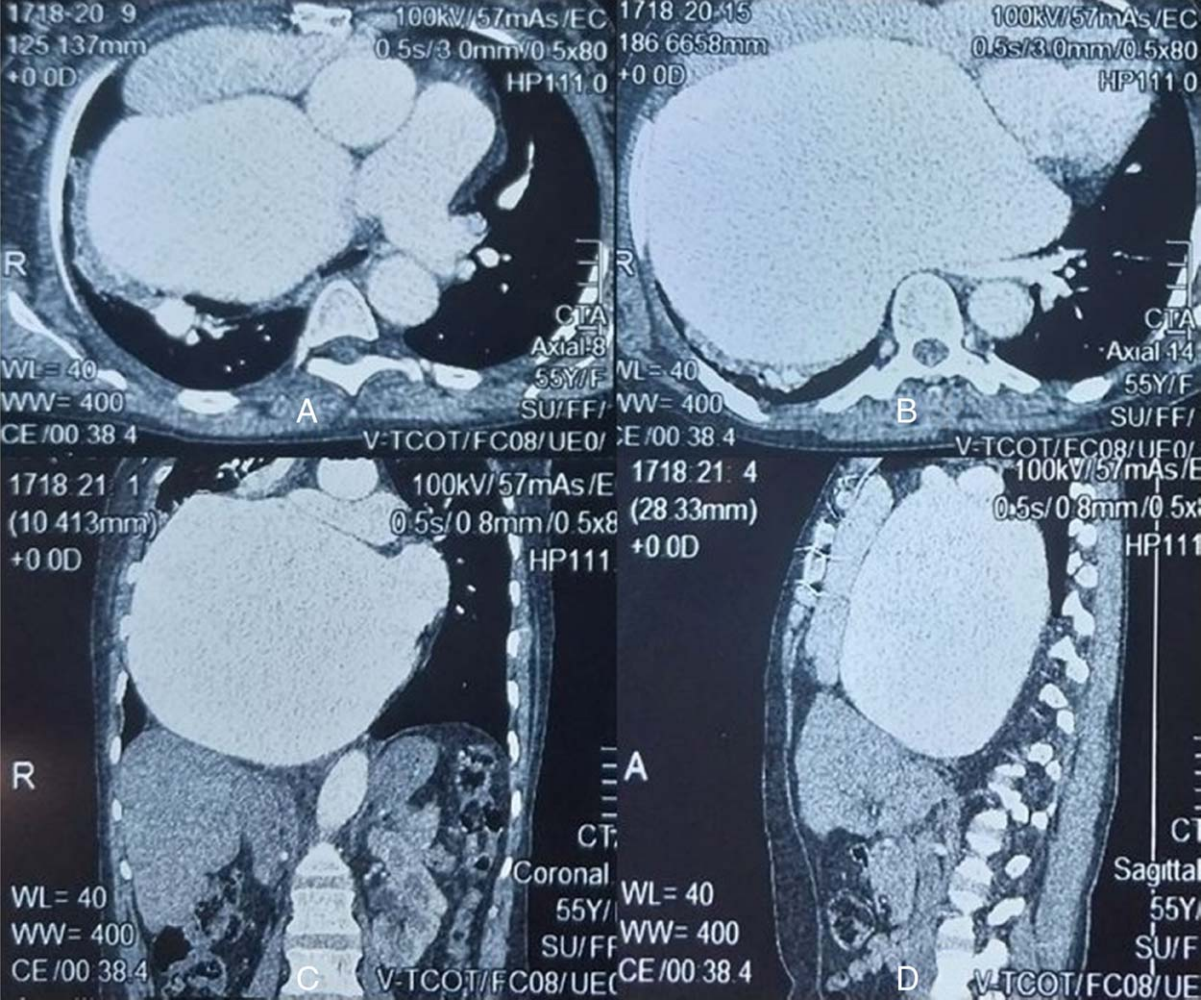
Computerized tomography angiography showed an enlarged lift atrium along with dilatation of pulmonary veins and an increase in the size of the pulmonary trunk giving a mosaic appearance of lung parenchyma (signs of pulmonary hypertension).

The chest X-ray results indicated cardiomegaly and pleural effusion on the right side (Fig. 1). The electrocardiogram showed atrial fibrillation, ventricular extrasystole, and left axis deviation (Fig. 2). The transthoracic echocardiogram revealed a significantly enlarged left atrium measuring 93 mm in the parasternal long-axis view and a dilated ascending aorta measuring 42 mm (Fig. 3). Severe pulmonary hypertension was also observed, with a Vmax of 2.5 and alteration of right ventricular systolic function. The mitral valve was severely stenosed and regurgitated, with an SM (surface mitrale) of 1.5 cm, SOR (surface de l’ouverture de régurgitation) of 60 mm, and GM (gradient moyen) of 13 mmHg. Additionally, there was severe tricuspid regurgitation with a Vmax of 3.23 m/5 and PAPS (pression artérielle pulmonaire systolique) of 51 mmHg. Further radiological investigations were conducted to evaluate the extent of the enlarged left atrium, and a CT angiography of the chest revealed marked dilatation of the left atrium, causing compression on the surrounding structures (Fig. 4). Signs of pulmonary hypertension were also observed, as the right lung showed dilatation of pulmonary veins and an increase in the size of the pulmonary trunk by 37 mm. This condition was responsible for the mosaic perfusion of the lung parenchyma, but there was no evidence of thromboembolic events.

The laboratory investigations revealed the following results: the white blood cell count was 7500/ml, hemoglobin level was 10.5 mg/dl, and the platelet count was 347 000/ml. The fasting blood glucose level was 90 mg/dl, and the urea level was 0.24 g/l. The creatinine level was 8.6 mg/l, and the CRP (C-reactive protein) level was 35 mg/l. The prothrombin time was 21 s, and the international normalized ratio (INR) was 3.65. The partial thromboplastin time was 37 s.

During the patient’s hospitalization, they were treated with diuretics and ACE inhibitors. After discharge, they were stabilized on a medication regimen that includes captopril twice a day, lasix, and anticoagulant Sintrom. The patient is required to attend regular follow-up appointments every month to adjust the dosage of diuretics and anticoagulants according to their physical symptoms and INR, respectively.

## Discussion

The exact etiology of GLA is not fully understood. Many authors attribute it to carditis secondary to rheumatic heart disease, which causes the weakening of the left atrial wall. Other studies have shown that genetic mutations may contribute to remodeling of left atrial enlargement. To define GLA, the enlargement of the left atrium should exceed 65 mm – in our case, the left atrium was 93 mm^5^.

Long-standing mitral valve disease, whether stenosis or regurgitation, may contribute to a GLA, which is considered a compensatory mechanism for increasing pressure or volume overload of the atrium. This compensatory mechanism aims to reduce pulmonary venous congestion and pulmonary hypertension, which eventually leads to right-sided heart failure^6^.

GLA can be associated with many complications, either cardiac or extracardiac. Cardiac complications include atrial fibrillation, pulmonary congestion, and pulmonary hypertension, which are particularly prominent in this patient and are thought to be responsible for severe dyspnea and palpitations. There is no evidence of extracardiac complications such as compression of the esophagus causing dysphagia or compression of the left recurrent laryngeal nerve causing hoarseness (cardiovocal syndrome, Ortner’s syndrome)^5,7^.

Specific considerations are kept in mind concerning the management of this rare case, especially in the lack of medical resources. Conservative treatment and surgery are both involved in the management plan^8^.

There are many debates in this area. In clinical practice, many doctors treat GLA conservatively until significant disability occurs, such as right-sided heart failure or there are extracardiac complications. However, all authors agree that the appearance of any complications, either cardiac or extracardiac, is the main indication for surgery^9,10^. They claim that reducing left atrial wall size will reduce pressure effects with a favorable effect on the postoperative course. This will also reduce left atrium volume, which will prevent recurrent thrombosis by reducing intra-atrial stasis. Some authors claim that just the presence of GLA is an indication for surgery, even if the patient is asymptomatic^11,12^. This opinion is made based on observations made following the maze procedure, which focuses mainly on thromboembolic complications as it is more common and more dangerous. They concluded that the long-term success rate of the maze procedure and its modifications depend on the size of the left atrium^13^. Chen *et al.* showed that preoperative left atrial size and left atrial diameter (cut-off value of 56.25 mm) could predict successful conversion to sinus rhythm. More than 90% of the patients with a preoperative left atrial size <57.8 mm regained sinus rhythm postoperatively^14^.

Kosakai^15^ showed that when the left atrium diameter was <45 mm, atrial fibrillation was ablated in 100% of the patients, but when the diameter was >85 mm, the success rate was 0%^15^. Most surgeons believe that successful mitral valve surgery alone will result in the eventual reduction of left atrial size as the volume and mean atrial pressure decline. In our opinion, this is not true. One major reason is that the changes observed in the left atrium of patients with GLA are quite considerable and sometimes irreversible. Hagihara *et al*.^16^ reported the early and long-term surgical results and changes in the left atrial dimension among 30 GLA patients. They observed that the diameter of left atrium decreased significantly from 69.0±8.5 mm to 53.7±9.1 mm shortly after surgery and maintained at 5 years of follow-up. As shown by these studies, the reduction in size is not significant and probably does not alleviate the symptoms caused by GLA. The most important point to note here is that despite mitral valve surgery, the enlarged left atrial size is unlikely to reduce in size without direct surgical management of GLA.

The coexistence of GLA and mitral valve disease increases mortality from 8 to 23%. In the modern era, mortality more than 5% is not accepted because of modern anesthesia and postoperative care. According to Piccoli *et al*.^17^ increasing operative mortality is proportionate to high pulmonary systolic pressure.

The current methods for left atrial volume reduction may be classified into three categories: partial plication or excision of the inferior atrial wall, partial plication or excision of both inferior and superior atrial walls, and partial autotransplantation of the heart. Nonetheless, all of these procedures will reduce mortality. The intended benefits of reducing GLA include elimination of symptoms and alleviating pressure symptoms of GLA; reduction in early postoperative complications related to low cardiac output and respiratory complications; and an increase in the probability of regaining sinus rhythm^5^. Scherer *et al*.^11^ showed in a comparative study that GLA reduction after radiofrequency ablation contributes to a higher restoration of sinus rhythm (77.3 vs. 61%) after 1 year.

## Conclusion

The quality of the observational data cannot justify any strong recommendations to our patients especially when mortality can be high. Cardiovascular surgeons should carefully consider the contemporary benefits of the various surgical options when managing GLA, screening and follow-up are essential for patients with long-standing rheumatic heart diseases for early management and detection of GLA.

## Ethical approval

Not applicable.

## Consent

Written informed consent was obtained from the patient for the publication of this case report and accompanying images. A copy of the written consent is available for review by the Editor-in-Chief of this journal on request.

## Sources of funding

There are no funding sources.

## Author contribution

All authors were involved in the work’s conception and design, paper writing, article revision, and final revision and approval.

## Conflicts of interest disclosure

The authors declare that they have no conflicts of interest.

## Research registration unique identifying number (UIN)

1. Name of the registry: Research Registry.
2. Unique identifying number or registration ID: researchregistry9123.
3. Hyperlink to your specific registration (must be publicly accessible and will be checked): https://www.researchregistry.com/registernow#home/registrationdetails/6480e9683995660026aa068f/.

## Guarantor

Echreiva Med Sidi El Moctar.

## Data availability statement

Not available.

## Provenance and peer review

Not commissioned, externally peer-reviewed.
